# Numerical validation of the applicability of the simplified ventricular model in the analysis of hemolysis in the mitral paravalvular leak

**DOI:** 10.3389/fbioe.2025.1714076

**Published:** 2026-01-12

**Authors:** Krzysztof Truchel, Krzysztof Wojtas, Michał Marchel, Wojciech Orciuch, Łukasz Makowski

**Affiliations:** 1 Faculty of Chemical and Process Engineering, Warsaw University of Technology, Warsaw, Poland; 2 1st Department of Cardiology, Medical University of Warsaw, Warsaw, Poland

**Keywords:** computational fluid dynamics, dynamic mesh, hemodynamics, hemolysis, large deformation diffeomorphic metric mapping, mitral valve, paravalvular leak, shear stress

## Abstract

In this paper, we explore various approaches to model the hemodynamic changes during cardiac contraction in the presence of a mitral paravalvular leak. Using computational fluid dynamics and large deformation diffeomorphic metric mapping, we conducted simulations that represented ventricular motion in four distinct ways. Taking tomography data into account, we developed a heart model that accurately reproduced the actual heart structure. Two simplifications for ventricular geometry to streamline the modeling process were proposed: a static mesh and a universal geometry. The simulation results from the most intricate variant, the CT-based, real model with dynamic mesh, were compared with the outcomes from the simplified approaches, universal geometry and static mesh. The simulations described unsteady flow dynamics during contraction, using a non-Newtonian Carreau-Yasuda blood rheological model. As expected, the hemodynamic conditions and parameter values derived from the hemolysis criterion (shear stresses exceeding 300 Pa) demonstrated no significant discrepancies between the various models under scrutiny. This suggests that the analysis of this phenomenon can be simplified to employ a static and universal ventricular mesh, eliminating the necessity for patient-specific medical imaging of the ventricle. Such a simplification can significantly reduce preprocessing and computational time, making this model more practical for routine medical diagnostics.

## 1 Introduction

For many years, cardiovascular diseases have persistently ranked among the foremost causes of mortality (Mensah et al., 2023). Heart failure (HF), a condition affecting over 3% of the population in Europe and North America, can be attributed among others to valvular heart disease (VHD) (Podlesnikar et al., 2018; Triposkiadis et al., 2022). Population-based studies indicate that the prevalence of VHD increases strongly with age from approximately 1% in individuals under the age of 45 to as high as nearly 14% in those aged over 75 years (Nkomo et al., 2006). Given the demographic shift toward an aging population, it is reasonable to anticipate a concurrent rise in the incidence of VHD in the coming years.

Among VHD, aortic stenosis and mitral regurgitation (MR) are the primary diseases (Nkomo et al., 2006). Based on the MIDA registry of MR cases with non-surgical interventions, the long-term survival rate remains at around 30% for the highest-risk subgroup (highest MIDA score) after 10 years. Meanwhile, patients with the highest initial risk score who opted for surgical interventions demonstrate a more favorable survival rate of 37%. Currently, surgical treatment of mitral valve disease mainly offers two alternatives: valve repair or replacement. Although the former shows higher efficacy and reduced mortality risk, unfortunately, it remains unfeasible in a significant number of cases (Acker et al., 2014). Consequently, over the past half-century, approximately four million valve replacement procedures have been executed, and it is projected that the annual number of such surgeries may rise to as many as 850,000 by the year 2050 (Sun et al., 2009).

One of the more common and serious complications after valve insertion is the incomplete apposition between the native valve annulus and the implant. This defect results in an artificial passage connecting the ventricle to the atrium, commonly called a paravalvular leak (PVL). The development of this channel enhances heart failure and can lead to increased hemolysis, characterized by the rupture of red blood cells (RBCs) (Cruz-Gonzalez et al., 2016). Notably, the emergence of PVL after artificial valve surgery is seen 7%–22%. Although 74% of PVL cases occur within the first year, PVL resulting from prosthetic valve dissection or endocarditis occurs later. Clinically significant PVL occurs in up to 2%–5% of all patients and can potentially cause symptomatic hemolytic anemia, congestive heart failure, and, if left untreated, death (Shah et al., 2019).

The population study that examined individuals with PVL (excluding patients with less than mild PVL and patients with prohibitive risk precluding reoperative cardiac surgery (RCS)) shows that after confirmation of PVL diagnosis, 71% of patients underwent RCS within a median of 8 days. All patients had symptoms of class ≥ II NYHA classification. In the subgroup of patients undergoing RCS due to mitral PVL, the average hospital stay was 18 ± 16 days. Periprocedural outcomes included 3% mortality, 16% postoperative shock requiring vasopressors, 33% transient atrial fibrillation, 8% renal failure requiring dialysis, and 17% advanced heart block requiring permanent pacemaker implantation. During long-term follow-up (6.6 ± 4 years), mortality was significantly higher in mitral PVL compared to aortic PVL (54% vs. 40%) (Shah et al., 2019).

In cases involving small leaks with low levels of hemolysis, conservative management is the primary approach, although a considerable proportion ultimately necessitates surgical or interventional procedures. Until recently, complex cases of PVL mainly required surgical intervention, involving either repair or replacement of the prosthetic valve. It is noteworthy that both surgical methods showed failure rates ranging from 12% to 35%, and re-intervention was associated with an increased risk of mortality (Bouhout et al., 2016). Nevertheless, for the past several decades, transcatheter PVL closure techniques have been continuously becoming more popular in clinical practice (King, 1976; Hourihan et al., 1992; Cruz-Gonzalez et al., 2016; Cruz-Gonzalez et al., 2017). It is worth noting, however, that to this date these methods have not yet received certification from the United States Food and Drug Administration.

According to the medical literature, one of the main indications for PVL closure is blood hemolysis (Cruz-Gonzalez et al., 2016). A successful procedure significantly reduces heart failure and the amount of hemolysis. Despite satisfactory echocardiographic results, for unclear reasons, increased hemolysis is observed in 19.7% of patients after percutaneous PVL closure (Smolka et al., 2016). A literature review did not find studies to determine the mechanism of hemolysis after PVL closure.

In the case of PVL, the process of hemolysis appears to be multifactorial. Many sources undertake analyses of the effect of fluid flow on erythrocyte destruction (Sutera and Mehrjardi, 1975; Jones, 1995; Klaus et al., 2002; Fraser et al., 2012). Shear stress and RBC exposure time to shear stress are usually reported as the two main parameters. This is as expected since stress is the parameter that determines the magnitude of shape deformation, which in turn reflects the magnitude of mechanical deformation. As elastic elements, RBCs are resistant to deformation to a certain range, but when the deformation is large enough and the rate of deformation is high, they can be ruptured. According to sources, viscous stresses observed in laminar flow are not the most likely to lead to increased hemolysis. However, when combined with turbulent stresses, such a phenomenon is already more likely (Klaus et al., 2002). Thus, in the analysis of flow hemodynamics, not only viscous stresses but also turbulent stresses should be taken into account.

It is also noteworthy that the Kolmogorov scale for cardiac blood flow conditions is most often comparable to or smaller than the RBC size, which means that turbulent energy dissipation and pressure fluctuations can also affect the mechanical rupture of the RBCs (Klaus et al., 2002). This is another hint that analyzing hemolysis based on viscous stress alone, without considering turbulence, would lead to underestimating its amount.

The existing literature includes many considerations for qualitative assessment of hemolysis. Many studies have attempted to determine the threshold value of shear stress in relation to exposure duration, beyond which the manifestation of hemolysis becomes apparent. This threshold value shows a wide range, extending from 150 Pa (for exposure durations of the order of minutes) (Alemu and Bluestein, 2007; Ge et al., 2008; Fraser et al., 2012; Wiegmann et al., 2018) to 400 Pa (Sallam and Hwang, 1984), and in some cases up to 800 Pa (Lu et al., 2001) (for exposure durations on the order of milliseconds). A comparative analysis of those studies suggests that exposure durations, especially for shorter exposure durations, have a limited impact on recognized threshold values (Klaus et al., 2002).

Furthermore, in addition to those critical values, many indicators in the literature derive from correlations between parameters such as shear stress and exposure time. Nevertheless, their application varies, and their limitations argue against their reasonable use in this field of research (Yu et al., 2017). In the context of the following study, it is necessary to note that the hemolysis indicator is only used to facilitate comparative analysis of different models of ventricular dynamics during systole. Therefore, the exact numerical value of this index is of secondary importance, as it primarily indicates the degree of similarity between different models. Due to the significant variability in proposed threshold values, this study took a critical value of 300 Pa, a threshold level that finds agreement in the literature (Nevaril et al., 1968).

Nowadays, analysis of hemodynamics is increasingly being carried out based on simulations using computational fluid dynamics (CFD). In the literature, several studies on the analysis of the pathogenesis of hemolysis in the heart, e.g., around the aortic valve (Alemu and Bluestein, 2007; Ge et al., 2008) or other mechanical elements of the heart, e.g., blood pumps (Wiegmann et al., 2018; Liu et al., 2019; Puentener et al., 2021; Gao et al., 2023) can be found. However, the exploration of hemolysis in the specific case of mitral paravalvular leak (PVL) appears to be a relatively uncharted domain in the extant literature.

There is a paper in the literature that looks at hemolysis in cases of mitral PVL (Garcia et al., 1996). It lists several characteristic flow behaviors through the PVL and correlates biochemical indicators of hemolysis with them. Notably, this pioneering work, complicated by the lack of precise imaging and computational limitations at the time, offers an insightful but not exhaustive study of the subject. It leaves unanswered questions about the underlying mechanisms governing the pathogenesis of hemolysis in mitral PVL.

Previous research conducted by our research team, as documented in previous publications (Wojtas et al., 2021; Kozłowski et al., 2022; 2021), has shown that the use of CFD for this pathological condition has broad potential and enables easy and quick analysis of hemolysis. However, the question arises whether the use of simplified heart models was valid. In the present study, we represented the movement of the ventricle during cardiac systole based on CT scan data. The results of CFD simulations for this model were compared with the proposed simplified ones, and based on this, the validity of the simplifications used in previous and subsequent studies with mitral PVL was confirmed.

## 2 Materials and methods

### 2.1 Reference geometry

Blood flow in the heart is realized by the pressure difference in the heart chambers and arteries; chamber contractions are responsible for those changes. To accurately represent the flow conditions in the heart, it is therefore necessary to recreate the heart’s movement as closely as possible. The analyzed problem of hemolysis at PVL takes place during left ventricular (LV) contraction when the aortic valve (AV) is open and the mitral valve (MV) is closed. Neither left atrial (LA) contraction nor aortic motion occurs then. It follows that the aorta itself, the LA, and the PVL can remain stationary in the simulations.

**FIGURE 1:**
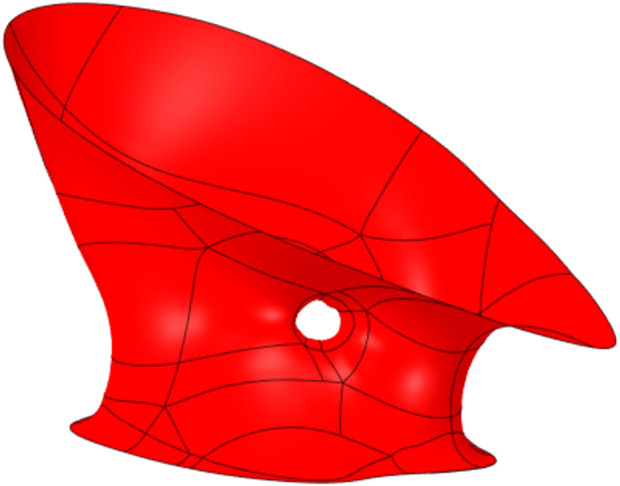
Geometry of PVL.

**FIGURE 2:**
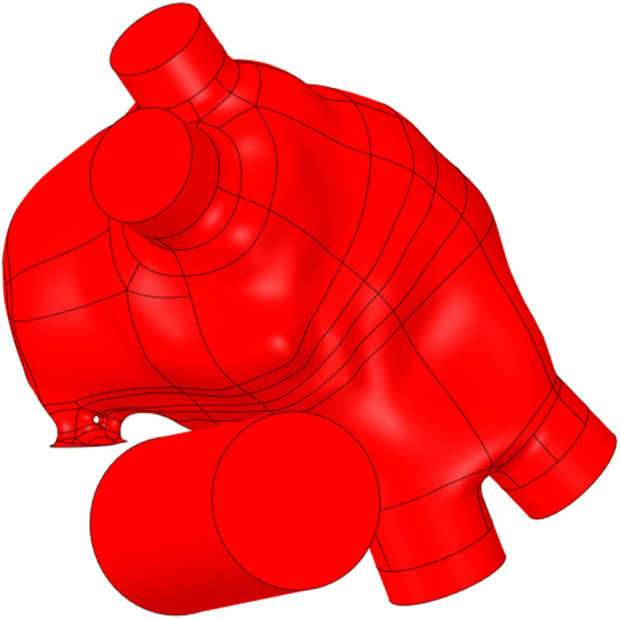
The stationary components of the reference geometry.

**FIGURE 3:**
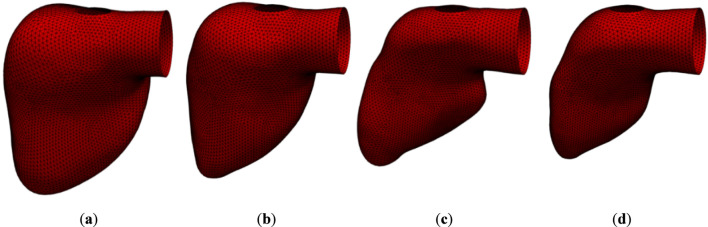
LV surface meshes at different moments of contraction of real geometry: **(a)** diastole; **(b)** 1/2 contraction; **(c)** 2/3 contraction; **(d)** systole.

The geometry of the LA with an attached PVL was derived from computed tomography (CT) scans and presented in previous stage of study (Kozłowski et al., 2022). PVL dimensions are presented in Table 1. Semi-automatic segmentation of the CT data was performed in the 3D Slicer software. The obtained image was then processed in ANSYS SpaceClaim 2025 R1 software to ensure it was free of any defects that could undermine CFD simulations. Blood vessels were added to the model based on their location in the tomograms and the average dimensions of an adult human, as outlined in the literature (Table 2) (Evangelista et al., 2010; Hassani and Saremi, 2017). The isolated PVL geometry can be seen in Figure 1 and the stationary components of the reference geometry can be observed in Figure 2 LV contraction in the reference geometry was mapped using CT images prepared similarly to the ones mentioned above. The CT images captured the shape of the LV at full diastole, complete systole, and two intermediate states, resulting in 4 tomograms. ANSYS Fluent 2025 R1 software was used to create four surface meshes based on these tomograms. These surface meshes were then modified using large deformation diffeomorphic metric mapping (LDDMM) (Fishbaugh et al., 2017) and the open-access Deformetrica software (Bône et al., 2018) to ensure that each mesh had the same number of nodes while retaining its original shape (Figure 3; Table 3). The geodesic regression method was used for this purpose, in which for each transition between two consecutive meshes, the optimal parameters must be determined (Bône et al., 2018): $\sigma_{\epsilon }$ (the average value of the magnitude of the deformation), $\sigma_{W}$ (the degree of match between the deformed object and the target object), and σ (the weight between the regular target mesh and the exact match to the output mesh). Throughout the study, it was noted that better mesh quality was achieved when the LDDMM deformations of the chamber mesh were modeled from contracted to expanded geometry (i.e., the opposite of the CFD blood flow simulations performed later). Additionally, the best mesh quality was obtained when the parameters $\sigma_{\epsilon }$ and $\sigma_{W}$ were equal to each other. Their exact values are shown in Table 4.

**TABLE 1:** Dimensions of mitral PVL in the plane of the smallest cross-sectional area.

Dimension	Canal I	Canal II	Whole PVL
Perimeter, (mm)	11.673	13.403	25.076
Cross-sectional area, (mm^2^)	10.663	12.109	22.772
Equivalent diameter, (mm)	3.654	3.614	3.632

**TABLE 2:** Dimensions of blood vessels.

Blood vessel	Diameter (mm)	Length (mm)
Aorta	25.00	50.00
Right superior pulmonary vein (RSPV)	17.40	5.00
Right inferior pulmonary vein (RIPV)	16.50	5.00
Left superior pulmonary vein (LSPV)	16.95	5.00
Left inferior pulmonary vein (LIPV)	14.45	5.00

**TABLE 3:** Ventricular volume at different moments of contraction of real geometry.

Moment of contraction	Volume (mm^3^)
(**a**) Diastole	173,803
(**b**) 1/2 contraction	122,935
(**c**) 2/3 contraction	81,943
(**d**) Systole	68,484

**TABLE 4:** Geodetic regression parameters for each LV grid transition.

Transition between meshes	$\sigma_{\epsilon }=\sigma_{W}$	σ
(**d**) Systole to (**c**) 2/3 contraction	5.0	0.001
(**c**) 2/3 contraction to (**b**) 1/2 contraction	8.0	0.001
(**b**) 1/2 contraction to (**a**) diastole	7.0	0.001

Knowing thus the position of individual nodes at 4-time steps from diastole to systole, a dynamic mesh was created using 3D spline interpolation during simulation in ANSYS Fluent 2025 R1 software, which determined the displacement vectors of individual nodes. The amount of deformation at each time step was determined to ensure that a change in LV volume resulted in a given volume mass flux, Q , which is described in a further subsection.

### 2.2 Simplified geometries

This paper proposes two simplifications to the left ventricular contraction model: replacing the real geometry for a particular patient with a simplified one and universal for all patients and replacing the dynamic mesh with a static one.

In the initial simplification proposal, it was assumed that the shape of the ventricle did not heavily influence the hemodynamic conditions within the leak. This allowed for replacing the patient-specific ventricle shape, obtained through medical imaging, with a universal and simplified geometry. Based on other scientific studies (Seo et al., 2013), a model was constructed that controlled several dimensions to produce the desired chamber volume. The ventricle is represented as a rotating ellipsoid with height H and width D , which at the top transforms into a cylinder of diameter d and height h , the edges of which are rounded. By constructing two such geometries with volumes equal to total diastole and systole, and using linear interpolation, a dynamic mesh can be created analogously for a CT–based ventricle.

If the assumption is that the shape of the ventricle does not significantly affect the flow conditions in the PVL, perhaps it is possible to go a step further and see if a dynamic mesh is necessary. In the second proposed simplification, the time-varying geometry of the ventricle was replaced by a time-constant geometry with a volume intermediate between full diastole and systole. The part of the lateral surface of the ventricle that was most deformed during contraction was extracted as the inlet of the time-varying mass flux, Q , that is, analogous to that induced by the change in volume of the ventricle.

**FIGURE 4:**
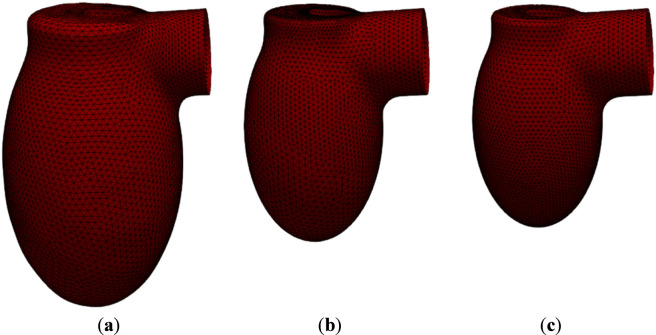
LV surface meshes at different moments of contraction of universal geometry: **(a)** diastole; **(b)** 2/3 contraction; **(c)** systole.

To assess the feasibility of implementing the proposed simplifications in future scientific and medical research, we compared the simulation outcomes of the reference geometry ((**I**) real geometry, dynamic mesh) with the individual simplified models: (**II**) universal geometry, dynamic mesh; (**III**) real geometry, static mesh; (**IV**) universal geometry, static mesh. The shape and dimensions of the universal geometries of the ventricle with volumes for the diastolic and systolic phases (for (**II**)) and for 2/3 systole (for (**IV**)) are presented in Figure 4 and Table 5.

**TABLE 5:** Ventricular volume and dimensions at different moments of contraction of universal geometry.

Moment of contraction	$\left.d\right.$ (mm)	$\left.h\;\right.$ (mm)	$\left.D\;\right.$ (mm)	$\left.H\;\right.$ (mm)	$\left.V\;\right.$ (mm^3^)
(**a**) Diastole	28.50	6.20	55.50	94.50	172,865
(**b**) 2/3 contraction	28.50	14.25	37.50	65.00	81,688
(**c**) Systole	28.50	14.25	20.25	55.75	68,135

### 2.3 Numerical method

CFD simulations of the transient internal blood flow in the four geometries were conducted using ANSYS Fluent 2025 R1 software. The analyzed medium, blood, was described as a homogeneous fluid with a constant density of ρ = 1060 kg m^−3^ (Wang et al., 2001). Due to the non-Newtonian shear-thinning properties, the viscosity of blood was described by the Carreau-Yasuda equation (Boyd et al., 2007), which is widely used as a rheological model for blood (Boyd et al., 2007; Mezali et al., 2023; Ali et al., 2015; Jędrzejczak et al., 2023a; Qayyum et al., 2023; Kozłowski et al., 2021; 2022; Wojtas et al., 2021):

$$\mu \left(\dot{\gamma }\right)=\mu_{\infty }+\left(\mu_{0}-\mu_{\infty }\right){\left(1+{\left(\lambda \dot{\gamma }\right)}^{a}\right)}^{\frac{n-1}{a}},$$

where the values of each constant are given in Table 6.

**TABLE 6:** Constants in the Carreau-Yasuda rheology model (Boyd et al., 2007).

$\mu_{0}\;($ Pa·s)	$\mu_{\infty }\;($ Pa·s)	a	n	$\lambda \;\left(s\right)$
0.1600	0.0035	0.64	0.2128	8.2

A three-dimensional balance of mass and momentum described the motion of the fluid:

$$\nabla \cdot \vec{v}=0$$

$$\rho \left(\frac{\partial \vec{v}}{\partial t}+\vec{v}\cdot \nabla \vec{v}\right)=-\nabla p+\nabla \cdot \tau$$

Due to the turbulent nature of blood flow in the PVL area and jet in the LA, the k−ω shear stress transport (SST) model with standard values of parameters proposed in ANSYS Fluent software, which are recommended for numerical calculations in physiological systems (Varghese and Frankel, 2003) and were used in the previous part (Kozłowski et al., 2021; 2022; Wojtas et al., 2021), was chosen to solve the above system of balance equations. The current model employed is founded on the Reynolds Averaged Navier-Stokes (RANS) method, whereby the closure problem of the averaged balance equations is resolved using the Boussinesq concept. In this approach, the Reynolds stress, $\tau^{R}$ , is expressed by means of a formulaic representation as follows:

$$\tau^{R}=-\rho \bar{{\vec{v}}^{\prime }{\vec{v}}^{\prime }}=\mu_{t}\nabla \bar{\tau }-\frac{2}{3}\rho kI,$$

where I is the unit matrix, and the turbulent viscosity, $\mu_{t}$ , and turbulent energy, k , are determined from the additional balance equations implemented by the k−ω SST model. The average value of the shear stress in the fluid, τ , is then given by the formula:

$$\tau =\left(\mu +\mu_{t}\right)\sqrt{2D_{ij}D_{ij}},$$

where $D_{ij}$ is the local fluid deformation rate:

$$D_{ij}=\frac{1}{2}\left(\frac{\partial v_{i}}{\partial x_{j}}+\frac{\partial v_{j}}{\partial x_{i}}\right)$$

### 2.4 Mesh

The influence of other differences between the geometries must be eliminated or minimized to make the comparison of the ventricle geometries themselves as unambiguous as possible. Therefore ensuring that the calculation mesh in each geometry is as similar as possible.

**FIGURE 5:**
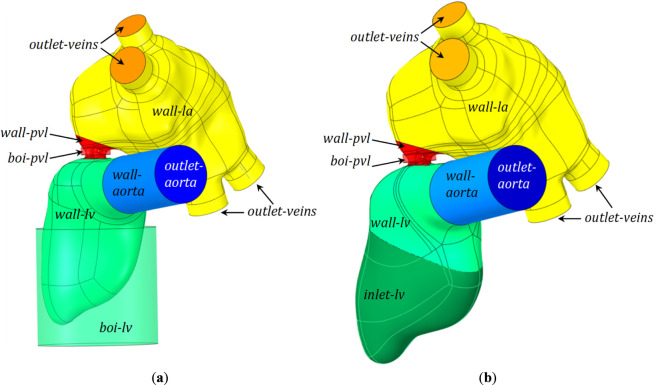
Left heart geometry with named surfaces and Body of Influence solids: green: wall-lv, dark green: inlet-lv, green transparent: boi-lv, red: wall-pvl, red transparent: boi-pvl, yellow: wall-la, dark yellow: outlet-veins, blue: wall-aorta, dark blue: outlet-aorta in **(a)** dynamic models ((**I**), (**II**)); **(b)** static models ((**III**), (**IV**)).

Using the geometries prepared in ANYS SpaceClaim 2025 R1 software (Figure 5a), volume meshes were generated in ANSYS Fluent Meshing 2025 R1 software for the dynamic meshes (models (**I**), (**II**)), keeping the same settings for each area. A tetrahedral mesh was used, ranging in size from 0.2 mm in the leakage area to a maximum of 3 mm in the ventricle volume, using local sizing such as Face Size and Body of Influence. Since hemolysis is predicted in the wall area, a 10-layer mesh densification has been added throughout the domain near the walls.

During simulations, the ventricular surface mesh was iteratively adjusted at each successive time step so that the change in ventricular volume induced the corresponding blood flow. A smoothing method was then used to create the volume mesh. The initial mesh expansion and the actual calculations for contraction were carried out using User Defined Functions (UDFs) compiled earlier in ANSYS Fluent 2025 R1.

The geometries of the static meshes (models (**III**), (**IV**)) were also prepared in ANYS SpaceClaim 2025 R1 software (Figure 5b). The cell size settings were identical to those for the dynamic meshes, except that part of the LV lateral surface was defined as an inlet, and no wall layer was generated on it.

**FIGURE 6:**
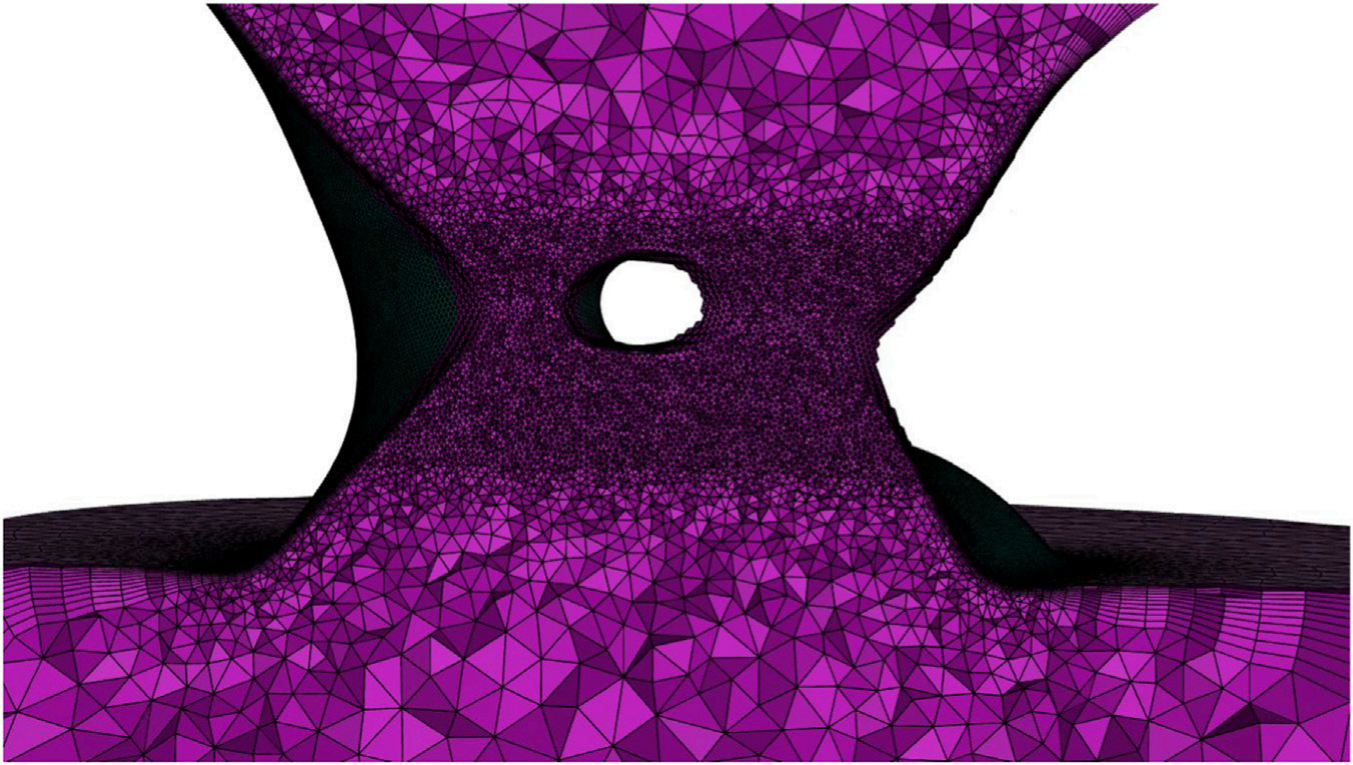
Final volume mesh in cross section view in the PVL area.

The effect of the number of cells on the obtained results was checked by comparing the average shear stress in the leakage area. Increasing the number of cells in the final mesh did not cause changes in these values above 2% (the impact was confirmed by the double thickening of the mesh in the volume of the leak and atrium). Table 7 shows the final number of cells obtained after this preliminary analysis of the impact of the grid and Figure 6 shows cross section of this volumetric mesh in neighborhood of the PVL.

**TABLE 7:** Number of cells in all studied cases.

Model	PVL surface cells	Total surface cells	Total initial volume cells
(**I**) Real dynamic	100,351	166,728	4,520 128
(**II**) Uni dynamic	104,033	169,274	4,581 660
(**III**) Real static	100,850	160,484	4,295 495
(**IV**) Uni static	104,409	163,502	4,354 301

### 2.5 Simulation setup

The cardiac pathology problem under analysis is characterized by time-varying hemodynamics. Consequently, the simulations were executed in a transient state with a time step of 0.02 ms. With the grid settings previously delineated, this time step guarantees sufficient convergence to satisfy the Courant-Friedrich-Lewy condition (Courant et al., 1928). In each time step residual of convergence of continuity and k−ω parameters reduced the value by more than 3-4 orders of magnitude, reaching at least a value of 10^–5^, which is considered a sufficient condition for convergence.

The contraction time was chosen to describe the moment of ventricular contraction at a resting heart rate of 60 bpm, for which the entire hemodynamic cycle takes 1 s. According to the findings of another research paper (Caballero et al., 2020a), the volumetric mass flow that will be forced by the change in volume of a dynamic grid or the inlet boundary condition of a static grid can be described by the following equation:

$$Q=-4\frac{Q_{\max}}{t_{\max}^{2}}{\left(t-\frac{t_{\max}}{2}\right)}^{2}+Q_{\max},$$

where the total time of systole phase, $t_{\max},$ was assumed as 0.36 s, and the value of maximum volume mass flux, $Q_{\max}$ , can be determined from the total volume change of the ventricle:

$$\Delta V=\int_{0}^{t_{\max}}Q\left(t\right)dt$$

For this case, $Q_{\max}$ is 4.388 ∙ 10^–4^ m^3^ s^-1^.

For static meshes, blood flow rate formula was defined as an inlet condition (*inlet-lv* in Figure 5b). In dynamic meshes (*wall-lv* in Figure 5a), knowing the coordinates of each vertex of ventricle surface mesh from time of whole diastole to whole systole with mid time stages (Figures 3, 4), the displacement curves of all nodes were calculated with cubic Hermite spline. The volume differential in a given time step was calculated as the integral of the above equation of blood flow over time. At the beginning of each time step the displacement of all vertex was calculated iteratively to satisfy the change of ventricle volume given by aforementioned formula with an accuracy of 10^–8^. After achieving the desired volume change, flow calculations began.

**FIGURE 7:**
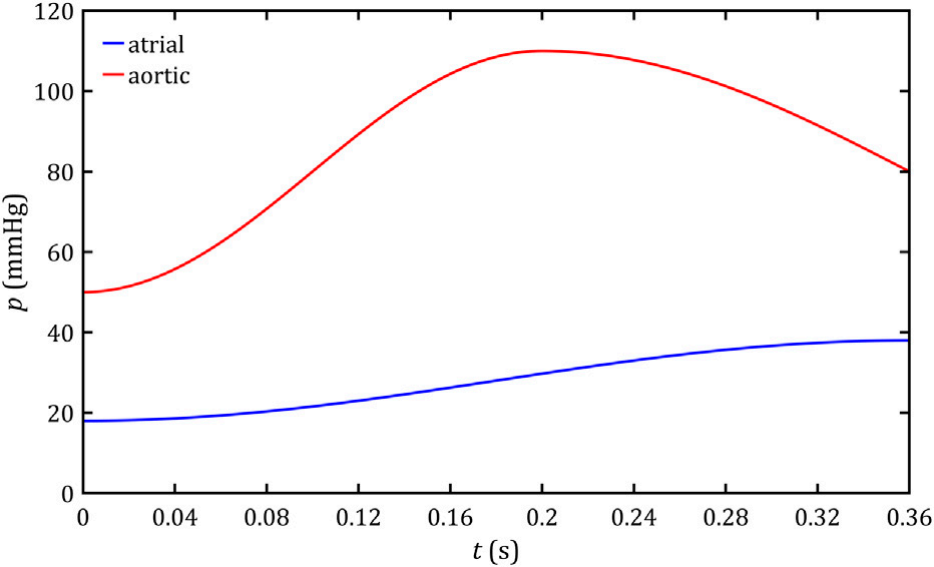
Atrial and aortic pressure profiles during the systolic phase.

Time-varying pressure profiles were selected as boundary conditions for outlets ( $p_{\text{LA}}$ for *outlet-veins* and $p_{\text{aorta}}$ for *outlet-aorta* in Figure 5). As indicated in the literature (Maor et al., 2017), the presence of a leak results in higher atrial pressures. Based on the reported real aortic and LA pressure waveforms in a patient with PVL (Maor et al., 2017), the shape of these profiles was mapped with high accuracy by approximating them with trigonometric functions. Employing the mean values of the characteristic points of the pressure waveforms for a group of patients with PVL reported in the literature (Lloyd et al., 2017) and considering the simplification of *c*-wave omission, which does not significantly affect the pressure profile in the heart in the analyzed case and is encountered in the literature when analyzing similar problems (Caballero et al., 2020b), the pressure profiles shown in Figure 7 and described by the formulas were obtained:

$$p_{\text{LA}}=0.5\;\cdot \left(p_{v-\text{wave}}-p_{a-\text{wave}}\right)\cdot \left(1-\cos\left(\pi \frac{t-t_{a-\text{wave}}}{t_{\max}-t_{a-\text{wave}}}\right)\right)+p_{a-\text{wave}}$$

$$p_{\text{aorta}}=\left\{\begin{array}{ll} 0.5\;\cdot \left(p_{\max}-p_{\min}\right)\cdot \left(1-\cos\left(\pi \frac{t}{t_{a-\text{wave}}}\right)\right)+p_{\min} & t<t_{a-\text{wave}}\\ 0.5\;\cdot \left(p_{\max}-p_{\min}\right)\cdot \left(1-\cos\left(\frac{\pi }{2}\frac{t-t_{a-\text{wave}}}{t_{\max}-t_{a-\text{wave}}}\right)\right)+p_{\min} & t\geq t_{a-\text{wave}} \end{array}\right.$$

where the values of each constant are given in Table 8.

**TABLE 8:** Mean values of pressure parameters for patients with PVL at a resting heart rate.

$p_{v-\text{wave}}\;\left(\text{mmHg}\right)$	$p_{a-\text{wave}}\;\left(\text{mmHg}\right)$	$t_{a-\text{wave}}$ (s)	$p_{\max}\;\left(\text{mmHg}\right)$	$p_{\min}\;\left(\text{mmHg}\right)$
38	18	0.2	50	110

## 3 Results and discussion

Due to the simplification of geometry modeling, which does not model the mechanical course of valve opening and closing, further analysis will be in the range of 0.04–0.32 s, due to the opening and closing time of both valves, which takes about 20–40 ms (Rubenstein et al., 1975; Zhong et al., 2021).

### 3.1 Blood flow and pressure

**FIGURE 8:**
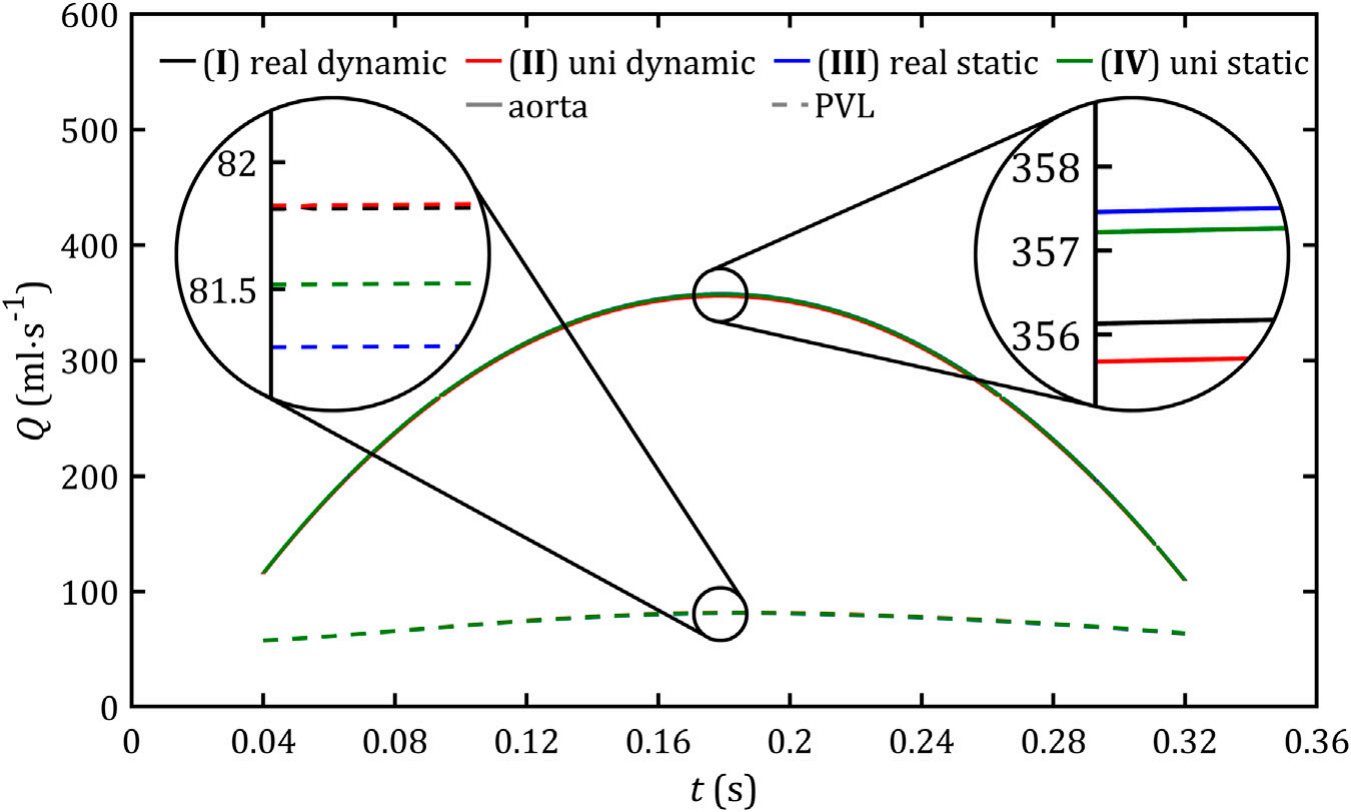
Blood mass flow through the aorta and PVL for all studied cases.

**FIGURE 9:**
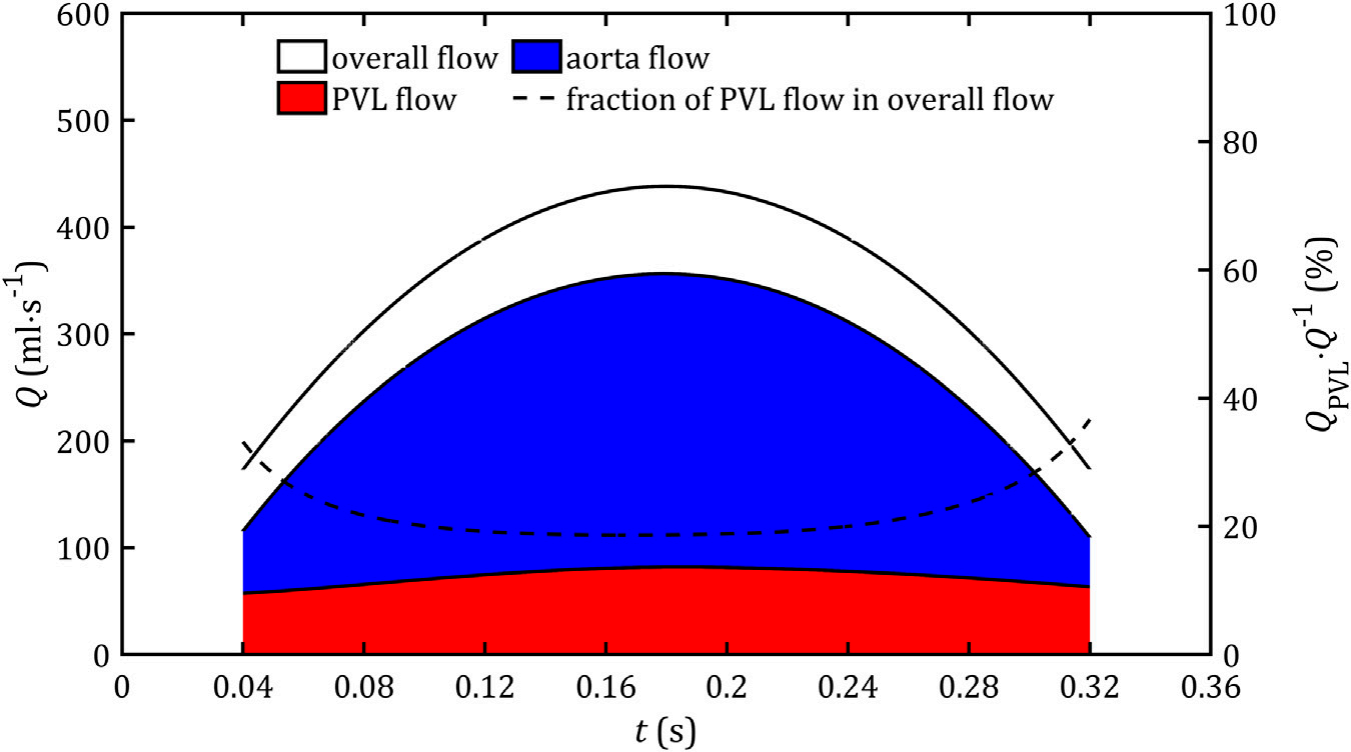
Blood mass flow through the aorta, PVL, total flow out of the ventricle, and its fraction that flows through the leak during the systolic phase.

The first important parameter that validates globally the applicability of the simplified ventricular model is blood flow. For the static domain, it is defined explicitly as a boundary condition, and for the dynamic one, it results from modeled deformations. Figure 8 shows comparison for flow through aorta and PVL for all studied cases. For all cases analyzed, the results were consistent, and the differences on the graph were negligible. Due to the minor differences Figure 9 presents the comparison of the whole blood flow through the entire heart and its part which flows through the leak based only on a reference model.

**FIGURE 10:**
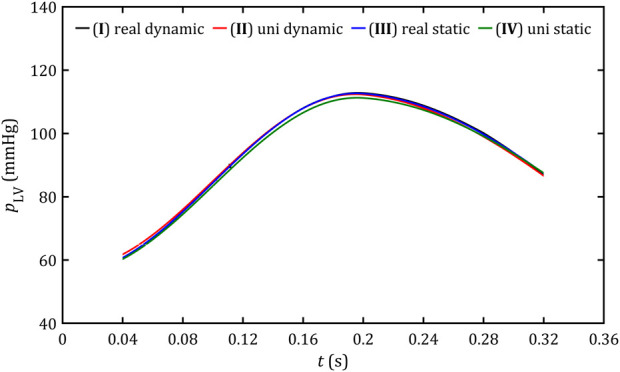
Profiles of mean pressure on the left ventricle wall for all studied cases.

As can be seen in Figure 9, the part of blood flow, which does not flow through the aorta (as it should be in a healthy heart), stays between 20% and 40% of the total heart flow, and its magnitude remains almost constant for the entire systole phase (range: 58–82 mL s^-1^). The relative errors for all simplified cases compared to real geometry and dynamic mesh are less than 0.5%. Similar observations could be noticed on pressure profiles in the left ventricle (Figure 10), which, for all cases, change in the range 60–112 mmHg and lead to the relative errors less than 1%.

### 3.2 Velocity fields and hemolysis regions

**FIGURE 11:**
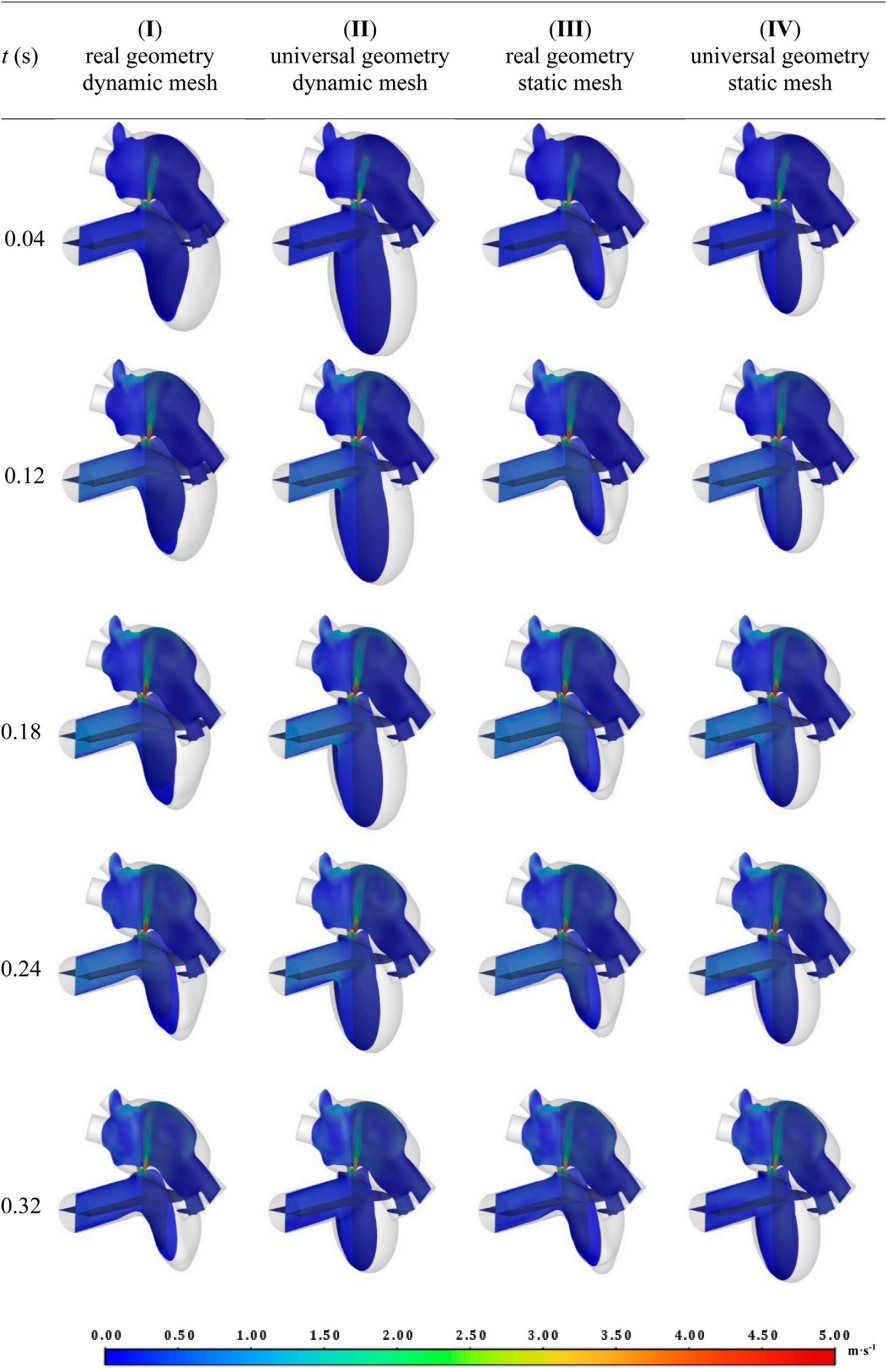
Velocity fields for chosen timepoints during the systole for all studied cases.

**FIGURE 12:**
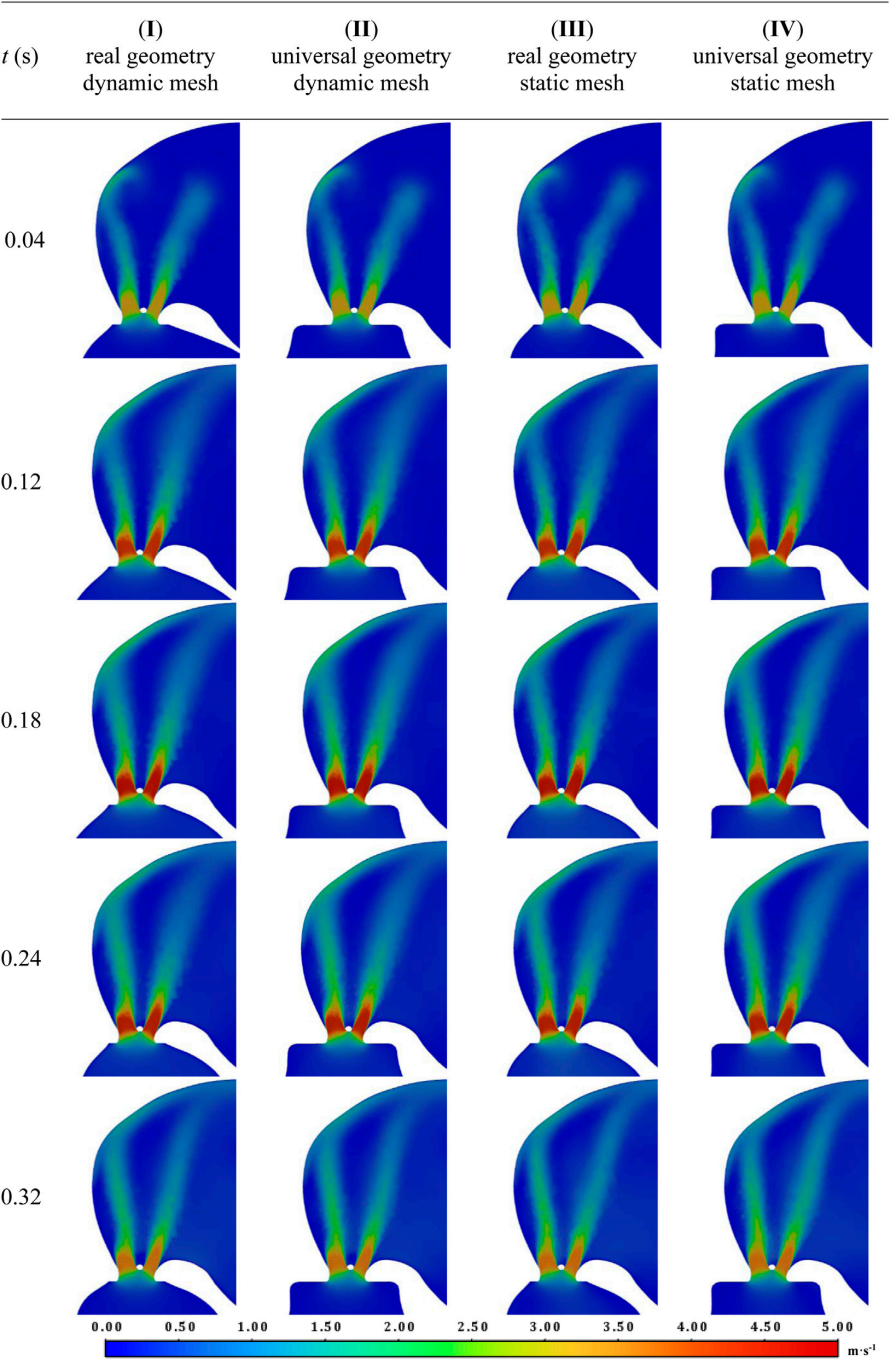
PVL and LA velocity fields for the chosen timepoints during the systole for all studied cases.

Visual analysis of velocity fields shown in Figures 11, 12 also provides a solid basis for considering consistency between the different approaches (in the Supplementary Material animations of changes in velocity fields for all studied cases are available). Highly convergent results were obtained within the leak and atrium for all selected time points and studied approaches. For all cases areas of high velocity are observed in both leak channels almost in the entire volume. For the analyzed geometry, two flame-like velocity jets form from the beginning of systole and reach the upper LA wall in its first moments. This is likely the direct cause of the observed increase in LA volume in patients with PVL (Kirali et al., 2001). This shape of the jets indicates high values of the velocity derivative at their boundary, which suggests that hemolysis is likely to occur there. The maximum observed velocity is slightly below 5 m s^-1^, which is consistent with the velocity measured in other PVLs in human hearts with similar cross-sectional areas (Choi et al., 2021).

**FIGURE 13:**
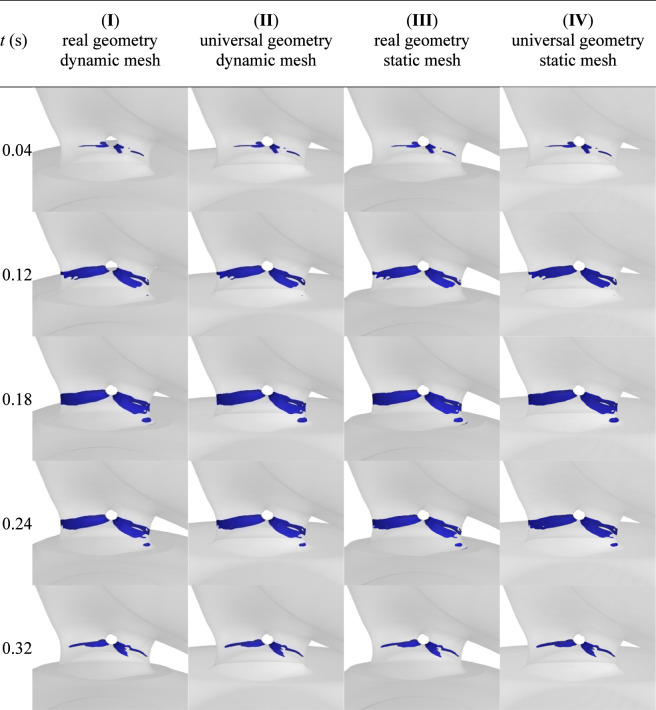
Regions of critical shear stresses for chosen timepoints during the systole phase for all studied cases.

Based on the applied threshold value of 300 Pa for shear stresses, the regions where hemolysis occurs were marked. Figure 13 shows these regions for selected time points (in the Supplementary Material animations of changes in regions of critical shear stresses for all studied cases are available). Hemolysis occurs during the whole systole phase only in the PVL zone. In the double-channel PVL, it takes place near the wall in the region with the largest constriction of the leak on the ventricular side. It begins and ends near the wall between the PVL channels, and in the middle phase of systole, it covers the entire circumference of the channels. These regions are visually very similar for all studied cases, and the aforementioned observations are valid for all approaches.

### 3.3 Hemolysis severity parameters

Further analysis will be based on specific parameter values related to shear stress. Since hemolysis occurs in the blood volume, but mainly in the wall space, the analysis is carried out using the volume with critical stresses ( $\tau_{V}$ > 300 Pa), $V_{300}$ , and the external wall area with critical stresses ( $\tau_{A}$ > 300 Pa), $A_{300}$ .

**FIGURE 14:**
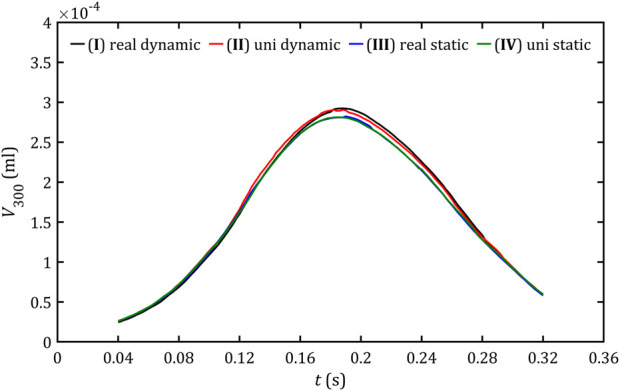
Volume of fluid with critical stresses for all studied cases.

**FIGURE 15:**
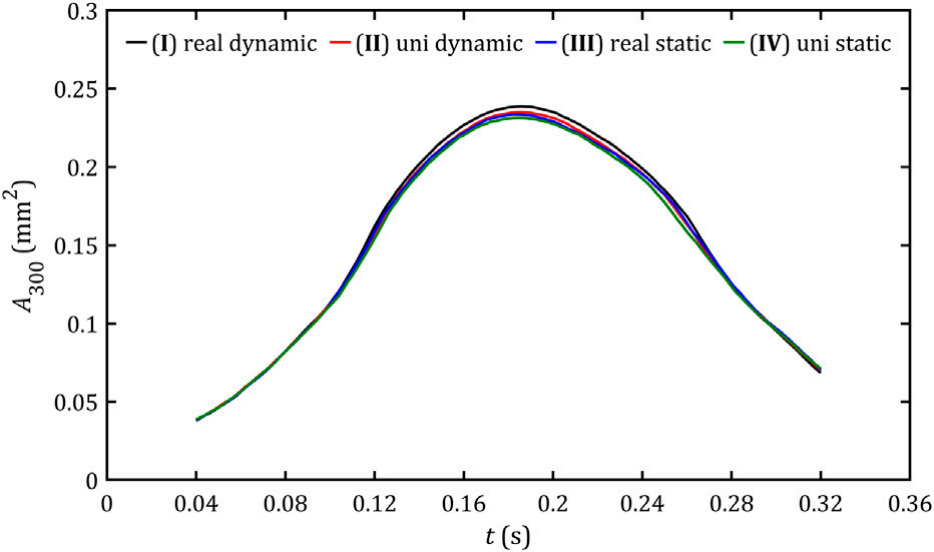
Area of PVL wall with critical stresses for all studied cases.

Both trends of changes in critical volume, $V_{300}$ , and critical area, $A_{300}$ , presented in Figures 14, 15 have a similar shape of an asymmetrical bell curve with a maximum at a point close to the maximum flow and a steeper slope for the increasing flow stage. Those trends are similar in different cases. For both parameters, dynamic meshes lead to slightly higher values.

**FIGURE 16:**
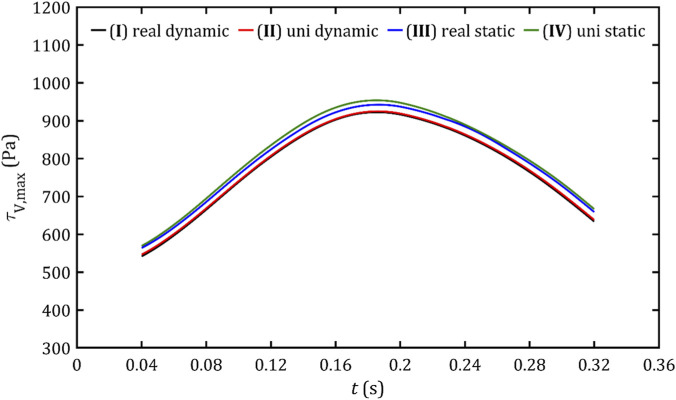
Maximum volume shear stresses in fluid volume for all studied cases.

**FIGURE 17:**
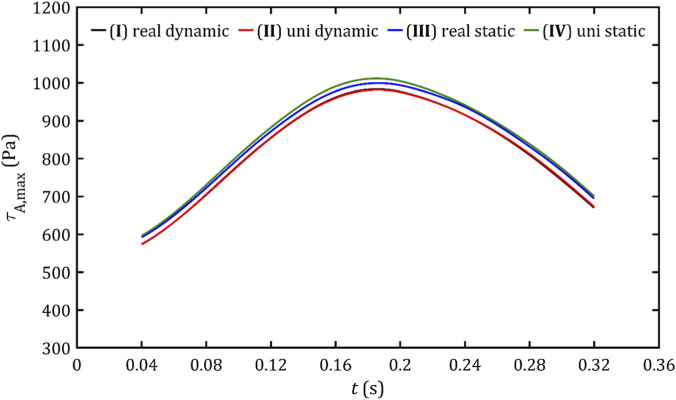
Maximum wall shear stresses in the PVL wall area for all studied cases.

In case of maximum shear stresses in fluid volume, $\tau_{V,\max}$ , and PVL wall area, $\tau_{A,\max}$ , presented Figures 16, 17 graphs exhibit a hill-like shape, with a maximum at the point of peak flow and a rise that has a steeper slope than the decrease. The difference between these and the previous graphs is that the increase and decrease of these parameters is approximately linear, and slightly lower values are obtained from dynamic meshes.

**FIGURE 18:**
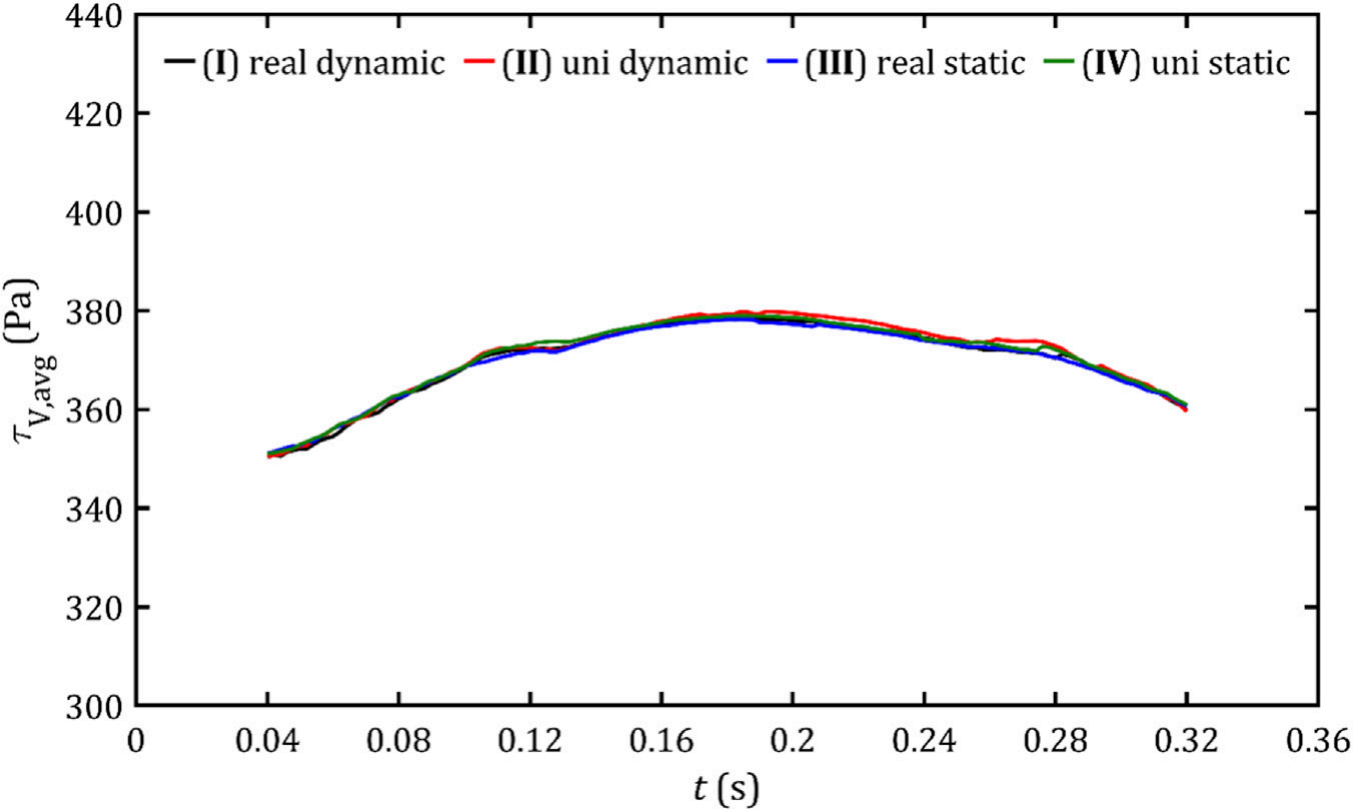
Average volume shear stresses in the critical volume for all studied cases.

**FIGURE 19:**
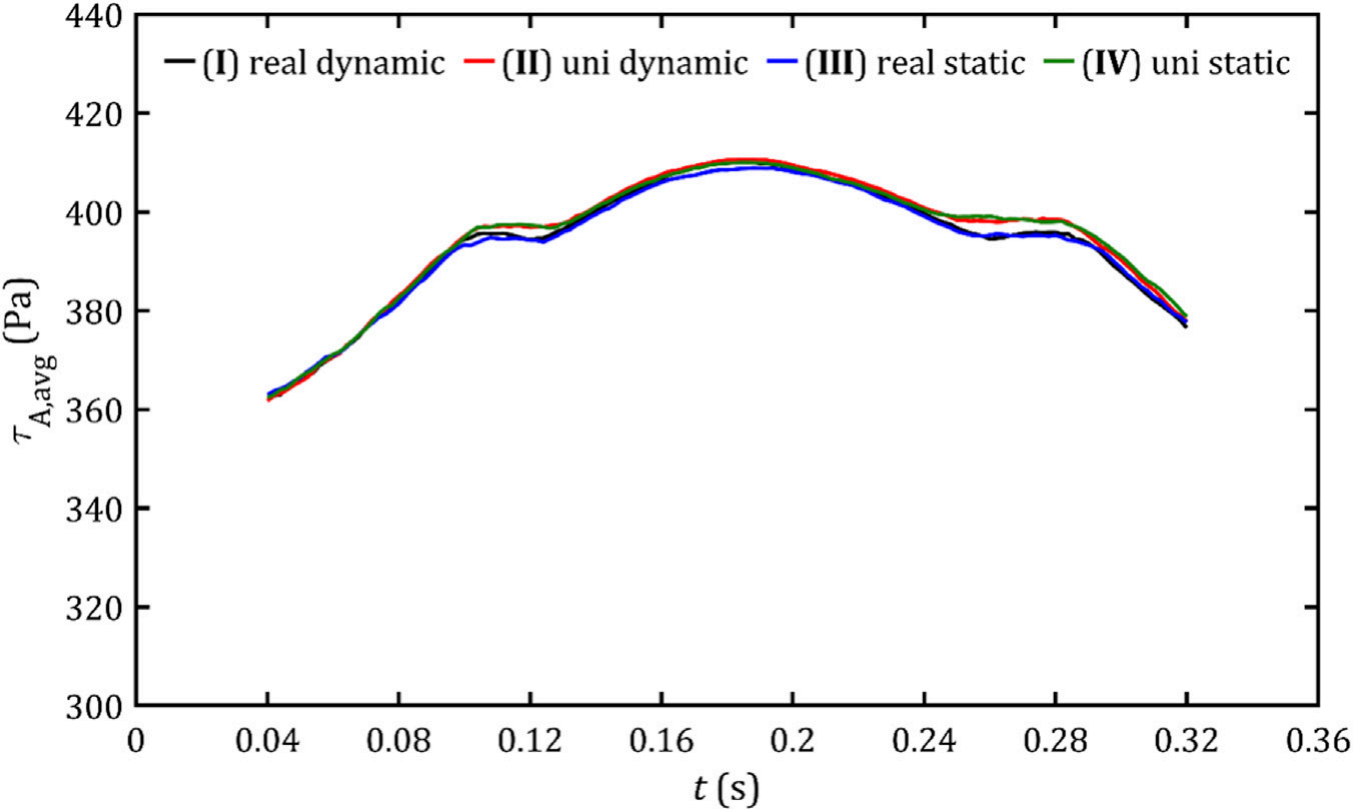
Average wall shear stresses in the critical area for all studied cases.

Despite the very similar maximum stress results, it is worth noting the average stress values in both volume and surface critical regions, presented in Figures 18, 19. These parameters allow the risk of hemolysis to be estimated at specific points where stresses are most significant and to determine the overall risk of erythrocyte rupture.

The waveform of both parameters looks like a three-peak hill, although for volume stresses, the curve is much smoother than for surface stresses. The shape seems to be a result not only of the flow and pressure gradient, but also of the leak geometry (the first and third peaks occur when the critical area begins and ends along the entire circumference of the channels). The values range from approx. 350–380 Pa (average 380 Pa) for in-volume stresses and 360–410 Pa (average 395 Pa) for wall stresses. This indicates that, within the range of critical stresses, the volume and surface of these stresses exhibit a distribution of values extending from the limit of 300 Pa to a maximum value. However, this distribution is not symmetrical, with the mean value tending to align more closely with the lower end of the spectrum. However, the hemolysis limit value adopted based on the literature significantly exceeds not only locally at the maximum value areas but also globally. Another important observation is that the average stresses remain almost the same throughout the entire ventricular systole. The approaches obtained here exhibit high similarity, except for minor areas, primarily peaks. Apart from these exceptions, the individual approaches demonstrate no significant visual differences.

In addition to the magnitude of the stresses and the size of the region where critical stresses occur, exposure time is also essential. For a closed region such as $V_{300}$ , assuming fluid incompressibility makes it difficult to determine the volume flow rate accurately. However, it can be estimated by determining the average fluid velocity weighted by the volume of the computational cells:

$$u_{avg}=\frac{1}{V_{300}}\sum_{i:\tau_{i}\geq 300\text{Pa}}\left|u_{i}\right|\cdot V_{i}$$

and referring to critical volume, $V_{300}$ :

$$t_{300}=\frac{\sqrt[{3}]{V_{300}}}{u_{avg}}$$

**FIGURE 20:**
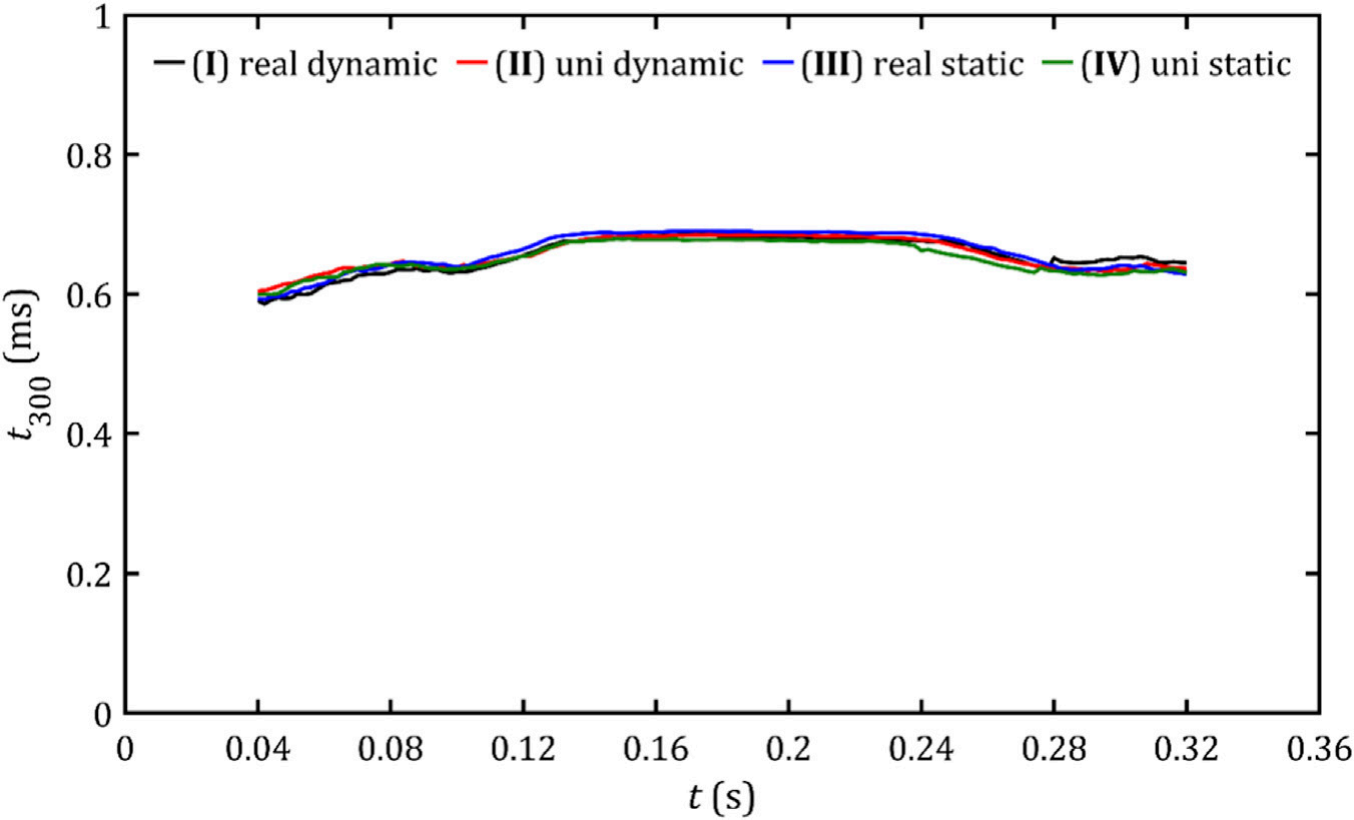
Estimated value of the residence time of the fluid in the critical volume for all studied cases.

Figure 20 shows changes in the estimated value of residence time during systole. They are consistent for all studied cases. Notably, for the analyzed geometry, this time is almost constant during the whole contractions and lasts approximately 0.6 ms.

The previously discussed graphs demonstrate a high degree of visual similarity between the reference and simplified models. The mean error, ε , and standard deviations, σ , presented in Table 9 indicate that all the simplifications analyzed are acceptable. The relative error values, ε , remain below 4.0% for $V_{300}$ , $A_{300}$ , $\tau_{V,\max}$ , $\tau_{A,\max}$ , $t_{300}$ and below 0.4% for $\tau_{V,\text{avg}}$ and $\tau_{A,\text{avg}}$ , irrespective of the model, thereby thus ensuring the validity of results obtained from all approaches in modeling cardiac hemodynamics in PVL. Dynamic modeling with a universal mesh across all models results in the lowest relative error values. While not having to prepare a per-patient mesh certainly simplifies the process and saves research time, it should be noted that dynamic modeling still involves high computational demands and thus long computation times. Using a static mesh with the actual geometry significantly reduces calculation and geometry preparation time. However, it leads to errors nearly identical to those of a static mesh and universal geometry. With the same solver settings, the calculation time for the universal mesh was significantly shorter, and the only geometry preprocessing required was cutting out the leak fragment and pasting it into the universal mesh. This approach is the least time-consuming and computing-power-intensive in geometry processing and calculations, and the simplest in geometry preparation. This shortens the response time from medical imaging of the patient’s heart to determining the extent of hemolysis through simulation. It also allows for creating an application that does not require experience in geometry processing or computational fluid dynamics. Furthermore, reducing the time required for calculations and domain preparation enables testing different process conditions, geometries, and leak locations. These tests can serve as the basis for developing a hemolysis risk map or creating a machine learning–based application that can estimate a patient’s risk of hemolysis without performing CFD calculations.

**TABLE 9:** Mean and standard deviations of relative errors of analyzed parameters for simplified cases (**II**–**IV**) in comparison to (**I**) real geometry and dynamic mesh.

Model	$\varepsilon_{V_{300}}\;\left(\%\right)$ ( $\sigma_{V_{300}}\;\left(\%\right)$ )	$\varepsilon_{A_{300}}\;\left(\%\right)$ ( $\sigma_{A_{300}}\;\left(\%\right)$ )	$\varepsilon_{\tau_{V,\max}}\;\left(\%\right)$ ( $\sigma_{\tau_{V,\max}}\;\left(\%\right)$ )	$\varepsilon_{\tau_{A,\max}}\;\left(\%\right)$ ( $\sigma_{\tau_{A,\max}}\;\left(\%\right)$ )	$\varepsilon_{\tau_{V,\text{avg}}}\;\left(\%\right)$ ( $\sigma_{\tau_{V,\text{avg}}}\;\left(\%\right)$ )	$\varepsilon_{\tau_{A,\text{avg}}}\;\left(\%\right)$ ( $\sigma_{\tau_{A,\text{avg}}}\;\left(\%\right)$ )	$\varepsilon_{t_{300}}\;\left(\%\right)$ ( $\sigma_{t_{300}}\;\left(\%\right)$ )
(**II**) Uni dynamic	2.572 (1.716)	1.411 (0.687)	0.424 (0.166)	0.156 (0.118)	0.226 (0.158)	0.307 (0.213)	0.986 (0.945)
(**III**) Real static	3.312 (1.746)	1.581 (0.841)	2.793 (0.557)	2.329 (0.583)	0.139 (0.095)	0.169 (0.088)	1.269 (0.428)
(**IV**) Uni static	3.248 (2.054)	2.413 (1.412)	3.931 (0.568)	3.339 (0.495)	0.166 (0.111)	0.295 (0.299)	1.312 (0.957)

### 3.4 Limitation

The study has several limitations. Due to the lack of accurate medical data on the patient from whom the CT scans were taken (time course of hemolysis levels or pressures, flows, or velocities in individual heart chambers), it is impossible to easily confirm the simulation results. However, a comparison of, for example, maximum velocities is consistent with other medical studies. However, such precise confirmation is not necessary in these studies because these simulations aimed to investigate possible modeling simplifications by comparing simulation results rather than determining the extent of hemolysis.

It is also worth noting that the residence time is only an estimate. Accurately determining would require a Computational Fluid Dynamics-Discrete Element Method (CFD-DEM) model, which requires a much denser grid and much longer calculations. This makes the method impractical for medical diagnostics. Another possible approach is using a population balance model, which adds balance equations describing erythrocytes' agglomeration and destruction processes to the flow equations. This approach, which has been prepared and tested by our team (Jędrzejczak et al., 2023b), will be applied in the subsequent phase of the research. In this phase, a comprehensive analysis of the severity and course of hemolysis will be carried out for universal geometry and static mesh, the applicability of which has been confirmed in these studies.

Another significant limitation is the selection of the hemolysis threshold value. Although the threshold of 300 Pa is commonly reported in the literature, it is not specific to PVL and should not be interpreted as physiologically definitive. Existing experimental benchmarks for predicting hemolysis (e.g., standard nozzle or blood pump tests) demonstrate significant variability based on stress definitions, turbulence models, and rheological assumptions. This variability limits the ability to claim precise, absolute accuracy for any hemolysis measure based on thresholds without calibration specific to the device or condition. In this study, the threshold was used solely as a parameter required to enable consistent comparison of modeling approaches. Due to the lack of an experimentally calibrated, PVL-specific hemolysis model, a value had to be selected based on the literature to perform the analysis. It is important to note that the current simulations were never intended to determine the absolute magnitude of hemolysis. Studies conducted for alternative threshold values (150 Pa and 400 Pa) showed that the relative trends between the compared modeling strategies remained unchanged, confirming the comparative nature of the intended study. The next stage of the research is to develop a calibrated hemolysis model specific to PVL. In this phase, we will combine the population balance approach with experimental data to provide a more realistic estimate of erythrocyte damage, eliminating the need for a threshold value. The results of this study allow us to apply the RBC population balance model to a static, universal model. This would be virtually impossible to implement for dynamic geometry due to the difficulties in model implementation and the high computational power requirements.

## 4 Conclusion

This article examines various approaches to modeling hemodynamic changes during ventricular contraction in cases of mitral paravalvular leak. The study aimed to compare a model based on computed tomography results, which accurately reproduced the actual structure of the heart and ventricular motion, with simplified approaches to ventricular modeling: a static mesh and simplified universal geometry. The comparative analysis examined regions with shear stresses exceeding the accepted limit value of 300 Pa, above which blood hemolysis occurs, which is detrimental to the patient.

Simulations using computational fluid dynamics (CFD) and large deformation diffeomorphic metric mapping (LDDMM) showed that it is possible to model left ventricular contraction using a dynamic mesh and the simplification to a static mesh with an inlet flow. A literature review also suggested eliminating the need for precise chamber geometry by using a simple, universal geometry for all patients. In the case of mitral paravalvular leak (PVL), both simplifications lead to results that do not differ significantly from those obtained through precise mapping of ventricular motion (high visual agreement of results and less than 4% relative error for parameters related to hemolysis). The simplified LV model can therefore be used as a basis for estimating hemolysis if the local geometry of the PVL channel is accurately represented and the physiological inlet conditions of the specific patient are applied. These simplifications proved to be accurate, and the significant reduction in the time and difficulty of preparing the geometry (from over a dozen hours of processing several CT scans to several minutes of processing the leak itself) and the reduction in calculation time (approx. 4 times) suggests that they are suitable for further analysis of hemolysis in PVL and other valve hemodynamics studies.

Additionally, simplifying the preparation of the geometry and adopting a simplified, universal approach to the calculations will enable the entire process to be automated in the future. This could provide the basis for developing a research methodology based on an application that does not require knowledge of fluid dynamics or the software. Further work using simplified geometry will investigate how various parameters (e.g., heart rate, stroke volume, pressure, leak location and size) affect the severity of hemolysis. Statistical analysis of those results could enable clinicians to draw conclusions that can serve as a basis for a preliminary assessment of the significance of the leak, even without any application.

## Data Availability

The raw data supporting the conclusions of this article will be made available by the authors, without undue reservation.

## Supplementary material

SUPPLEMENTARY VIDEO S1 Critical volume in real dynamic.

SUPPLEMENTARY VIDEO S2 Critical volume in uni dynamic.

SUPPLEMENTARY VIDEO S3 Critical volume in real static.

SUPPLEMENTARY VIDEO S4 Critical volume in uni static.

SUPPLEMENTARY VIDEO S5 Velocity field in real dynamic.

SUPPLEMENTARY VIDEO S6 Velocity field in uni dynamic.

SUPPLEMENTARY VIDEO S7 Velocity field in real static.

SUPPLEMENTARY VIDEO S8 Velocity field in uni static.
